# Efficacy and safety of mucous fistula refeeding in preterm infants: an exploratory randomized controlled trial

**DOI:** 10.1186/s12887-023-03950-1

**Published:** 2023-03-29

**Authors:** Eun Sun Lee, Ee-Kyung Kim, Seung Han Shin, Young Hwa Jung, In-Gyu Song, Yoo-Jin Kim, Hyun Young Kim, Young-Hun Choi, Kyung Chul Moon, Bohyun Kim

**Affiliations:** 1 Department of Pediatrics, Seoul National University Children’s Hospital, 101, Daehak-Ro, Jongno-Gu, Seoul, 03080 South Korea; 2 Department of Pediatrics, Seoul National University College of Medicine, Seoul, Republic of Korea; 3 Department of Pediatrics, Chung-Ang University Hospital, Chung-Ang University College of Medicine, Seoul, Republic of Korea; 4 Department of Pediatrics, Seoul National University Bundang Hospital, Seongnam, Republic of Korea; 5 Department of Pediatrics, Korea University Medical Centre, Guro Hospital, Seoul, Republic of Korea; 6 Department of Pediatrics, Chungbuk National University Hospital, Cheongju, Republic of Korea; 7 Department of Pediatric Surgery, Seoul National University Children’s Hospital, Seoul, Republic of Korea; 8 Department of Radiology, Seoul National University Children’s Hospital, Seoul, Republic of Korea; 9 Department of Pathology, Seoul National University Children’s Hospital, Seoul, Republic of Korea

**Keywords:** Necrotizing enterocolitis, Enterostomy, Citrulline, Short bowel syndrome, Randomized controlled trial, Preterm infants, Mucous fistula refeeding

## Abstract

**Background:**

This study aimed to evaluate whether mucous fistula refeeding (MFR) is safe and beneficial for the growth and intestinal adaptation of preterm infants with enterostomies.

**Methods:**

This exploratory randomized controlled trial enrolled infants born before 35 weeks’ gestation with enterostomy. If the stomal output was ≥ 40 mL/kg/day, infants were assigned to the high-output MFR group and received MFR. If the stoma output was < 40 mL/kg/day, infants were randomized to the normal-output MFR group or the control group. Growth, serum citrulline levels, and bowel diameter in loopograms were compared. The safety of MFR was evaluated.

**Results:**

Twenty infants were included. The growth rate increased considerably, and the colon diameter was significantly larger after MFR. However, the citrulline levels did not significantly differ between the normal-output MFR and the control group. One case of bowel perforation occurred during the manual reduction for stoma prolapse. Although the association with MFR was unclear, two cases of culture-proven sepsis during MFR were noted.

**Conclusions:**

MFR benefits the growth and intestinal adaptation of preterm infants with enterostomy and can be safely implemented with a standardized protocol. However, infectious complications need to be investigated further.

**Trial registration:**

clinicaltrials.gov NCT02812095, retrospectively registered on June 6, 2016.

## Background

Premature infants have immature intestinal function and are prone to developing meconium plug syndrome, necrotizing enterocolitis, and spontaneous intestinal perforation [1, 2]. Surgical intervention is often required with the creation of an enterostomy and mucous fistula [3, 4]. When premature infants have a large stoma output volume, enteral feeding may be difficult. Moreover, such situations can lead to delayed weight gain, dehydration, and abnormalities of electrolytes, macro (carbohydrates, proteins, and lipids), and micronutrients (vitamins and minerals). Consequently, prolonged parenteral nutrition (PN) is required, which induces catheter-related infection, cholestasis, and intestinal mucosal atrophy [5]. To solve this problem, mucous fistula refeeding (MFR) was introduced by Puppala in the 1980s. MFR is the practice of collecting proximal ostomy effluent and reinfusing it into the distal mucous fistula. It can prevent atrophy of the distal bowel and promote fluid and nutrient absorption [6, 7]. In previous retrospective studies, MFR was proven as a safe technique that helped in promoting infant growth and discontinuing PN [7–10]. However, no prospective studies have yet evaluated the efficacy and safety of MFR. Therefore, we conducted an exploratory randomized controlled trial (RCT) to evaluate whether MFR is safe and beneficial for growth and intestinal adaptation of preterm infants with stoma formation.

## Methods

### Trial design

This study was conducted at the neonatal intensive care unit (NICU) of Seoul National University Children’s Hospital as a single-center, exploratory RCT with parallel enrollment between July 1, 2015 and November 11, 2019. The study was approved by the Institutional Review Board of Seoul National University Hospital (IRB No. 1407–193-601) and registered with the ClinicalTrials.gov registry (NCT02812095). The study followed the standards established by CONSORT. Written informed consent for the study was obtained from the parents of the preterm infants. Preterm infants born before 35 weeks’ gestation with enterostomy were eligible for enrollment. We excluded patients with congenital anomalies (e.g., congenital bowel obstruction and congenital megacolon), blind pouches, stricture and unstable vital signs. When patients achieved the full feeding levels (volume of enteral feeds > 120 mL/kg/day), those with enterostomy effluent volume ≥ 40 mL/kg/day were categorized as the high-output MFR group, and all infants in that group received MFR. The other patients whose enterostomy effluent was < 40 mL/kg/day were randomly assigned to the control or trial group (defined as normal-output MFR) at a 1:1 ratio, using a computer-generated allocation sequence. Parents and medical staff were not blinded to the group allocation. For all the patient groups, the general management proceeded in the same manner, except MFR. A distal loopogram was constructed before MFR was started, and upon identification of a stricture, the infant was excluded from the study. Even for infants in the control group, they were excluded if the requirement for MFR was identified according to the clinical course.

The safety of the study was assessed daily. The patients were monitored for the following adverse reactions: respiratory (hypoxemia, tachypnea), cardiovascular (hypotension, tachycardia, bradycardia); gastrointestinal (bilious gastric remain, abdominal distension, vomiting); skin erosion, stoma problems (prolapse, irritation, bleeding); and infections (culture-proven sepsis, wound infection). If an adverse reaction occurred, the neonatologist in the clinical team appraised the situation and temporarily halted the refeeding as necessary. In case of serious adverse reactions, the intervention was terminated.

As this exploratory trial aimed to generate data on MFR for the power calculation of the full-scale RCT, the convenience sample size consisted of all eligible infants in our institution for 4 years. Approximately 5–10 infants required enterostomies per Year, and 20 patients were selected as the adequate number of subjects, assuming a 10% dropout rate.

We collected patient demographic and diagnostic characteristics, clinical outcomes, colon diameter on loopogram before reanastomosis, and serum citrulline levels at 4 timepoints: just before refeeding, after 4 weeks, just before reanastomosis, and 12 weeks after reanastomosis. We also collected the biopsy specimens of the distal ileum at the stoma closure and evaluated the infants for any MFR-associated complications.

### Nutrition

After the surgery for enterostomy, all infants received PN. If the surgeon judged that the patient's bowel condition had improved (e.g.: reduced amount of nasogastric tube output, x-ray bowel gas pattern) and could receive enteral feeding, feeding was started. Bolus feeding (every 2–3 h) was performed routinely. Fortified breast milk, preterm formula, or hydrolyzed protein formula were used for enteral nutrition (EN). Feeding was started at 20 mL/kg, and if tolerable, the amount was increased by 20-30 mL/kg per day. PN was discontinued when infants reached the levels of full feeding (enteral feed volume > 120 mL/kg/day) and age-appropriate weight gain was achieved without PN supplementation.

### Intervention protocol: mucous fistula refeeding

MFR was started when infants reached full enteral feeding, and the enterostomy output volume was sufficiently collected. MFR was initially performed by the surgeon and continued by the NICU nurse. We collected the proximal stoma output in pouches every 8 h and delivered 50% of it into the distal fistula. Initially, MFR was performed three times, and when there were no side effects, 50% was administered six times every 4 h. From then on, 100% was applied every 4 h. We manually delivered the output to the distal mucous fistula through a 3- or 4-Fr Nelaton catheter over 5–10 min. We used a new catheter for each infusion and subsequently removed the catheter. MFR was performed until reanastomosis, unless there were serious side effects.

### Outcome

The primary outcome was the efficacy of MFR, indicated by the growth, change of colon diameter (assessed using loopograms), and serum citrulline level. Growth was presented as the z-score of length and bodyweight. The secondary outcomes were duration of PN, histological results of the distal ileum at stoma closure, and the safety of MFR. Experienced pediatric radiologists assessed the bowel diameter changes in the loopograms, and pathologists evaluated the degree of chronic inflammation and villous structure of the distal ileum at the stoma closure. The degree of chronic inflammation was classified as mild, moderate, or severe depending on the number of monocytes between crypts. The radiologists and pathologists were blinded to the clinical status of the patients.

### Statistical analyses

Statistical analysis was performed using SPSS 20.0 (IBM, Chicago, IL, USA). A chi-squared test was used to compare categorical data between groups, while a Mann–Whitney U test was used to compare quantitative data between groups. P-values were derived from ANCOVA adjusted for postmenstrual age (PMA) and body weight z-score at stoma formation. *P*-values < 0.05 were considered statistically significant.

## Results

### Study population

**Fig. 1:**
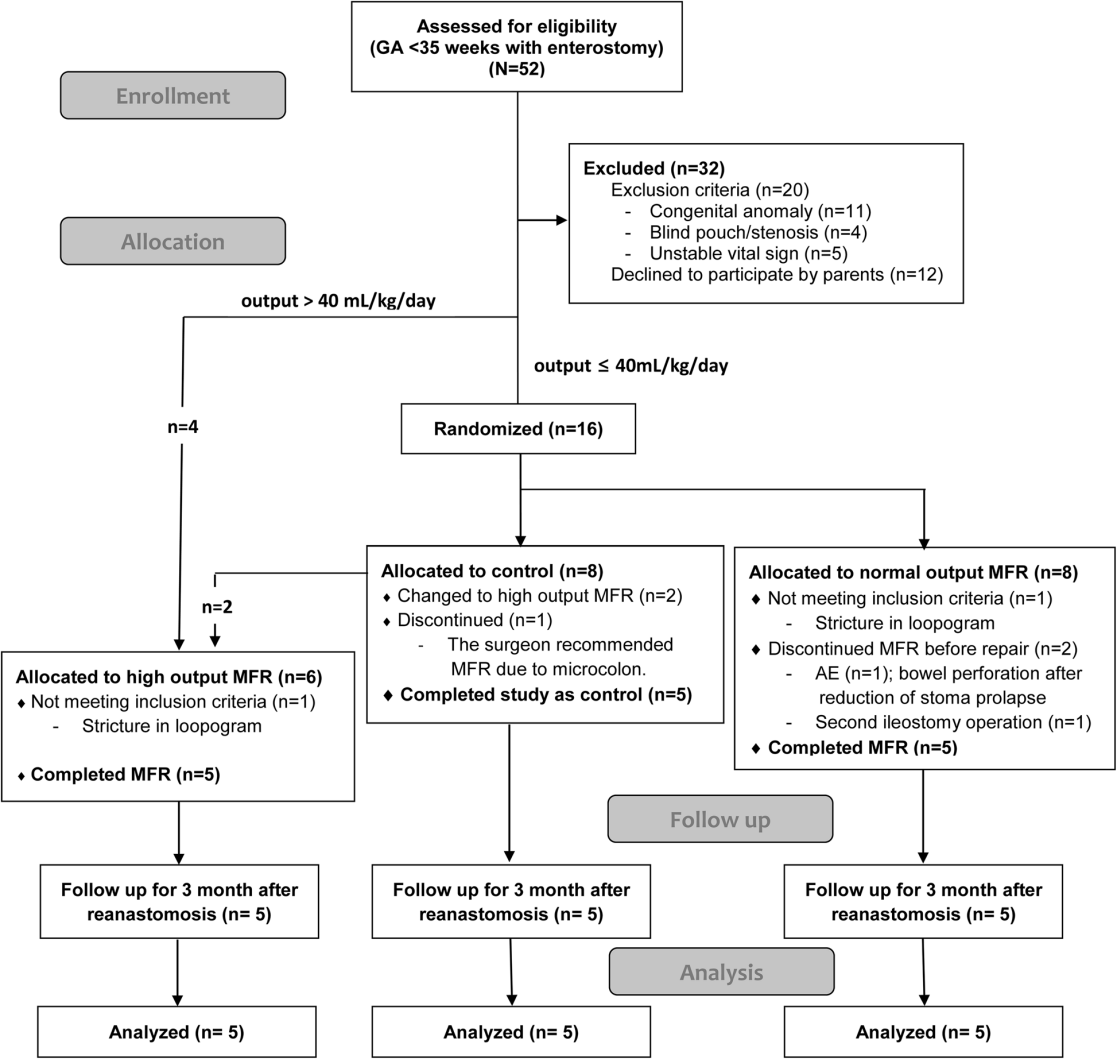
Flow diagram of the study. MFR, mucous fistula refeeding

A total of 52 infants born before 35 weeks of gestation who had enterostomies were eligible for the study. Of them, 32 infants were excluded per the exclusion criteria and the parents of 12 infants declined to provide consent. Therefore, 20 preterm infants were finally analyzed (4 infants allocated to the high-output MFR group before randomization; 16 infants were randomized to the control and normal-output MFR groups (*n* = 8 each). Two infants in the control group were reassigned to the high-output MFR group because their stoma output finally exceeded 40 mL/kg/day. Another infant in the control group started MFR based on the surgeon’s recommendation for micro-colon management. In the normal-output MFR group, three infants were excluded; in one case, a stricture was detected on the loopogram, another infant had a bowel perforation after stoma reduction following MFR, and another one required a second ileostomy (Fig. 1).

### Demographic and surgical information

The gestational age (25^+0^ vs. 26^+6^ vs. 29^+0^ weeks; high-output MFR vs. normal-output MFR vs. control) and birthweight (540 vs. 880 vs. 1,190 g) were lower in the high-output MFR group than that in the normal-output MFR and control groups (*p* < 0.05). However, the control and normal-output MFR groups showed no significant differences in the gestational age and the birthweight. The diagnosis and operative findings were not significantly different between groups (Table 1).

**Table 1:** Demographic and surgical information

	**High output** **MFR (*n* = 5)**	**Normal output MFR (*n* = 5)**	**Control (*n* = 5)**	***P*-value**^†^	***P*-value**^§^
**Gestational age (weeks)**	25 (23, 26 + 4)	26 + 6 (26 + 2, 33 + 5)	29 (27 + 4, 30 + 2)	0.013*	*0.151*
**Birth weight (g)**	540 (520, 620)	880 (620, 1240)	1,190 (850, 1,500)	0.028*	*0.310*
**Male**	2 (40)	2 (40)	4 (80)	*0.143*	*0.197*
**At stoma formation**					
Postnatal day	12 (6, 43)	13 (2, 28)	5 (1, 18)	*0.513*	*0.310*
Post menstrual age (week)	28 + 3 (24 + 5, 31)	28 + 1 (27 + 1, 37 + 5)	29 (27 + 4, 32 + 6)	*0.099*	*0.690*
**Diagnosis at stoma formation**				*0.241*	*0.223*
Necrotizing enterocolitis	1 (20)	2 (40)	2 (40)		
Meconium obstruction	2 (40)	2 (40)	0 (0)		
Spontaneous intestinal perforation	2 (40)	1 (20)	3 (60)		
**Jejunostomy**	1 (20)	1 (20)	0 (0.0)	*0.283*	*0.292*
**Preserved IC valve**	5 (100)	5 (100)	5 (100)	*1.000*	*1.000*
**Resection bowel length (cm)**	10 (0, 35)	10 (0, 30)	4 (0, 50)	*1.000*	*1.000*

Values are presented as the median (min, max), or number (%)

^**†**^ Control vs. MFR (Normal and High-output), § Control vs. MFR (Norma-output only)

*Abbreviations*: *MFR* Mucous fistula refeeding, *HCA* Histologic chorioamnionitis, *PROM* Premature rupture of membranes, *SGA* Small for gestational age, *RDS* Respiratory distress syndrome, *BPD* Bronchopulmonary dysplasia, *PDA* Patent ductus arteriosus, *IVH* Intraventricular hemorrhage, *PVL* Periventricular leukomalacia, *IC valve* Ileocecal valve

^*^ *p* < 0.05

### Clinical and laboratory outcomes

There was no significant difference in total PN duration from stoma formation to off-PN (50.5 vs. 44 days; normal-output MFR vs. control, *p* = 1.000). The z-scores of body weight at stoma formation in the normal-output MFR group were significantly lower than those in the control group (-1.79 vs. -0.43, *p* = 0.032). However at the time of reanastomosis, the Z-scores of the bodyweight (− 2.19 vs. − 3.82) and length (− 3.08 vs. − 4.33) were larger in the normal-output MFR group than in the control group. Similarly, the bodyweight (− 0.96 vs. − 2.98) and length (− 1.77 vs. − 4.05) z-scores at 3 months after reanastomosis were numerically larger in the normal-output MFR group than in the control group, without statistical significance. Colon diameter on loopogram before reanastomosis was significantly larger in the transverse (10.15 vs. 6.05 mm, *p* = 0.002), descending (10.22 vs. 6.06 mm, *p* = 0.005), and sigmoid (12.48 vs. 7.07 mm, *p* = 0.037) colon in the normal-output MFR group than in the control group. The citrulline levels did not significantly differ between the normal and control groups. However, the median value of the normal-output MFR group was higher just before reanastomosis (31.19 vs. 26.29 µmol/L) and at 12 weeks after reanastomosis (35.45 vs. 27.23 µmol/L) than those of the control group. The control group had more frequent severe chronic inflammation of distal ileum at the stoma closure (75% vs. 20%) (Table 2).

**Table 2:** Clinical and laboratory outcomes

	**Normal output** **MFR** **(*n* = 5)**	**Control** **(*n* = 5)**	***P*-value**^§^	***P*-value**^**¶**^
**Total days of MFR**	39 (17, 75)	0	*-*	
**Total PN duration after stoma formation to off-PN (days)**	50.5 (16, 118)	44 (32, 68)	*1.000*	
**Reach full feeding after reanastomosis (days)**	12 (7, 17)	8 (6, 9)	*0.111*	
**At reanastomosis operation**				
POD of enterostomy	103 (73, 123)	85 (64, 122)	*0.548*	
PMA	44 + 5 (37 + 4, 49 + 5)	42 + 2 (39 + 1, 45)	*0.421*	
**Body measurement (z-score)**				
*At stoma formation*				
Body weight	-1.79 (-2.69, -0.95)	-0.53 (-1.45, -0.10)	0.032*	
Length	-1.68 (-2.37, -1.18)	-0.43 (-1.62, 0.07)	*0.063*	
*At reanastomosis operation*				
Body weight	-2.19 (-4.53, -1.11)	-3.82 (-4.32, -3.36)	*0.690*	
Length	-3.08 (-4.46, 0.28)	-4.33 (-6.0, -3.94)	*0.421*	
*Three months after reanastomosis*				
Body weight	-0.96 (-2.01, 0.1)	-2.98 (-3.51, -2.07)	*0.841*	
Length	-1.77 (-2.38, 0.21)	-4.05 (-4.31, -2.60)	*0.690*	
*Change of Z-score from stoma formation to reanastomosis*				
Body weight	-0.735 (-3.58, 1.58)	-1.54 (-3.66, 1.71)	*0.556*	
Length	-1.28 (-3.28, 2.65)	-1.67(-4.04, 0.67)	*0.730*	
**Distal loopogram before reanastomosis, diameter (mm)**				
Transverse colon	10.15 (7.3, 14.4)	6.05 (5.8, 7.8)	*0.114*	0.002*
Descending colon	10.22 (6.9, 14.3)	6.06 (4.8, 6.6)	0.029*	0.005*
Sigmoid colon	12.48 (8.9, 15.9)	7.07 (6.6, 11.3)	*0.114*	0.037*
Rectum	18.20 (12.9, 21.0)	16.6 (12.6, 22.4)	*1.000*	*0.573*
**Citrulline (umol/L)**^**¶**^				
Just before refeeding	20.89 (12.47, 38.27)	21.03 (12.18, 22.30)	*0.690*	*0.063*
After 4 weeks	32.23 (24.91, 44.30)	30.12 (18.49, 41.66)	*0.413*	*0.598*
Just before reanastomosis	31.19 (22.04, 44.30)	26.29 (18.03, 40.00)	*0.421*	*0.249*
12 weeks after reanastomosis	35.45 (26.52, 37.29)	27.23 (24.50, 34.78)	*0.190*	*0.858*
**Biopsy at stoma closure (distal)‡**				
Chronic inflammation			*0.196*	
Mild	2 (40)	0 (0)		
Moderate	2 (40)	1 (25)		
Severe	1 (20)	3 (75)		
Intact Villous structure	1 (20)	0 (0)	*0.556*	
**Culture proven sepsis**	1 (20)	1(20)	*1.000*	

Values are presented as median (min, max), or number (%)

^§^ Control vs. Normal-output MFR

^**¶**^ Control vs. Normal-output MFR. The values were adjusted for postmenstrual age at reanastomosis and bodyweight z-score at stoma formation. Citrulline levels were adjusted for postmenstrual age at the time of sample collection and body weight z-score at stoma formation

^‡^ One biopsy sample of control group was missed

*Abbreviations*: *MFR* Mucous fistula refeeding, *POD* Postoperative days, *PMA* Postmenstrual age, *HC* Head circumference, *PNALD* Parenteral nutrition-associated liver disease

^*^ *p* < 0.05

In the entire MFR group’s longitudinal comparison, weight and length growth was significantly accelerated after MFR with or without adjustment for PMA. However, colon size differences on loopogram were not significant after adjusting for the PMA of loopogram (Table 3).

**Table 3:** Bowel diameter and growth velocity of the MFR group

**High + normal output MFR group (*n* = 10)**	**Before MFR**	**After MFR (Before stoma closure)**	***P*-value**	***P*-value**^§^
**Dimeter (mm) on loopogram**				
Transverse colon	6.45 (3.90, 8.20)	8.93 (7.26, 14.40)	0.031*	*0.865*
Descending colon	6.3 (3.80, 10.43)	9.54 (6.9, 14.30)	*0.077*	*0.806*
Sigmoid colon	7.60 (4.70, 10.40)	10.99 (8.85, 15.90)	0.031*	*0.751*
Rectum	14.30 (8.60, 17.10)	17.35 (12.90, 21.00)	*0.206*	*0.990*
**Velocity of growth**				
Weight (g/day)	9.53 (4.76, 15.82)	23.36 (18.00, 36.11)	0.000*	0.000*
Length (cm/day)	0.09 (0.00, 0.13)	0.15 (0.08, 0.22)	0.003*	0.016*
Head circumference (cm/day)	0.08 (0.04, 0.13)	0.075 (0.00, 0.14)	*0.541*	*0.588*

Values are presented as the median (min, max), or number (%)

*Abbreviations*: *MFR* Mucous fistula refeeding

^§^The values were adjusted for the postmenstrual age at each time point

^*^ *p* < 0.05

### Safety

The MFR intervention was discontinued for 3 infants in the normal-output MFR group temporarily due to bowel distension/ prolapse/ skin erosion around the stoma and resumed after symptom improvement. The MFR intervention was terminated for one infant in the MFR group after a severe adverse reaction was reported. This patient received MFR for 10 days and suffered from bowel perforation during the manual reduction for stoma prolapse, thus requiring termination of MFR. Though the intervention was not interrupted because of an unclear association with MFR, two episodes of culture-proven sepsis (associated with *Klebsiella aerogenes* and *Staphylococcus epidermidis*)occurred during the study period; one in the normal-output MFR group and another in the high-output MFR group respectively. While maintaining the stoma in situ, four more cases of culture-proven sepsis occurred (Table 4).

**Table 4:** Cases of culture-proven sepsis after enterostomy

Case No	group	Onset of sepsis	Days after starting MFR	Days after enterostomy	pathogens	Antibiotics use (days)	No. of sepsis
1	Normal output MFR group	Before MFR	N/A	13	Staphylococcus epidermidis	10	1
2	High output MFR group	Before MFR	N/A	25, 49	Methicillin-resistant Staphylococcus aureus	16, 14	2
3	Control group	N/A	N/A	7	Candida albicans	32	1
4	High output MFR group	During MFR	15	161	Staphylococcus epidermidis	18	1
5	Normal output MFR group	During MFR	15	109	Klebsiella aerogenes	19	1

## Discussion

This is the first prospective RCT examining the safety and efficacy of administering MFR to preterm neonates. Our study showed that MFR is beneficial for increasing the bowel diameter and growth rate. Moreover, citrulline levels tended to be higher in the MFR group than for infants in the control group, which was not statistically significant.

Premature infants are capable of intestinal growth and adaptation after bowel resection [11]. Higher volumes of ostomy output adversely affect the growth and the body fluid and electrolyte status. Previous studies show that a stoma discharge < 40 mL/kg/day is considered ideal [12, 13]. To manage this situation and promote intestinal adaptation, a previous study showed the usefulness of MFR for growth and PN discontinuation because it artificially maintains the bowel flow to help absorption [10, 14, 15]. Furthermore, MFR improved the tolerance for EN after reanastomosis [16]. Therefore, we classified the infants with an ostomy output > 40 mL/kg/day into the high-output group and performed MFR to them. In the comparison of the outcomes of the normal-output MFR group and control group with an ostomy output < 40 mL/kg/day, no significant differences were observed in the PN duration, number of days required to reach full-feeding after reanastomosis, and the timing of reanastomosis.

The z-score of bodyweight at the stoma formation in the normal-output MFR group was significantly lower than that of the control group; however, at the times of reanastomosis operation and three months after the reanastomosis, the z-scores of bodyweight were not significantly different, thus meaning that MFR facilitated weight gain.

In the loopograms taken just before reanastomosis, colon diameters were significantly larger in the transverse, descending, and sigmoid colon of infants in the normal-output MFR group than those in the control group. Lau et al. [8] reported that MFR could decrease the risk of anastomotic complication. In our study, the anastomosis procedure had to be rescheduled for one infant in the control group due to a bowel-end size discrepancy. The infant subsequently underwent MFR and reanastomosis 75 days later.

Histopathological findings of the distal ileum at the stoma closure showed that chronic inflammation and destruction of villous structures were more frequent in the control group than in the normal-output MFR group. Similar to our findings, Yabe et al. [9] reported that MFR helped increase intestinal mucosal thickness and maintain the villous structure of the distal ileum. Intestinal maturation and rehabilitation are reinforced by exposure to enteral nutrients and enterotrophic factors [17, 18], which can be simulated by MFR.

Serum citrulline is a non-protein amino acid that is synthesized from glutamine and glutamine-related components within enterocytes [19, 20]. Serum citrulline is a widely used marker in infants to evaluate length and absorption capabilities of the small bowel and prognosis for PN weaning [21, 22]. We compared serum citrulline levels between groups and found no significant difference between the normal-output MFR and control groups. The median citrulline level at 4 weeks, just before reanastomosis, and 12 weeks after reanastomosis tended to be higher in the normal-output MFR group. This may indicate that MFR has a persistent positive effect on bowel rehabilitation after reanastomosis.

The amount of weight gain in premature infants naturally increases as PMA advances. Therefore, we compared the growth velocity before and after MFR after adjusting for PMA, by which the positive impact of MFR on weight gain would be verified more appropriately. Both of the daily weight and length gain were significantly larger after MFR in high-output + normal-output MFR group after adjusting for PMA.

In our study, several complications had occurred. MFR was terminated in one case due to perforation during a manual stoma reduction and minor complications of stoma prolapse, bowel distension and skin erosion were reported. Haddock et al. [23] conducted a study on MFR using a 12-Fr catheter that constantly implanted and reported that 17% of patients who underwent MFR experenced serious complications including bowel perforation or bleeding. In recent studies evaluating the safety of MFR, several factors including catheter size, personnel responsible for insertion, and insertion method were standardized, and no major MFR-related complications (perforation, stricture, or death) were reported [7, 9]. By standardizing the protocol, a more refined process could be performed using a weight-appropriate catheter. During the MFR procedure, there were two cases of culture-proven sepsis, each from the normal-output and high-output group. However, the intervention was not discontinued because the direct relationship between MFR and sepsis could not be determined. Even without any intervention, four additional cases of culture-proven sepsis had occurred. The conditions of extremely low gestational age and the presence of a central venous catheter and stoma alone confer a very high risk for sepsis to this study population. In infants with enterostomies, the intestinal epithelial barrier function is decreased due to the mucosal inflammatory response and villous atrophy, which facilitate the movement of luminal bacteria and its constituents into the underlying tissue and blood, which increases the susceptibility of bloodstream infection [24]. Approximately 13% of recurrent sepsis cases occur as a complication in newborns with enterostomies [25]. Pataki et al. studied the microbiological safety of recycling bowel contents and reported that the stoma effluent was colonized by commensal facultative pathogenic enteral and skin flora including coagulase-negative *Staphylococcus* after 120 min [26]. The microorganisms cultured during MFR in this study were *Staphylococcus epidermidis* and *Klebsiella aerogenes*, which are stomal pathogens too. On the contrary, Yabe et al. cultured the stoma output 3 h after its ejection and detected no pathogenic bacteria [9]. In a recent review, infection was not described as a MFR-related complication [27]. In our study, stoma contents were collected and recycled every 4 h. Two cases of sepsis occurred during MFR, but the relationship between MFR and sepsis could not be determined in this study. Therefore, we ascertain the necessity to probe whether this recycling interval is associated with a risk of infection. Further research is necessary on the infectious complications in the future studies and careful clinical monitoring for infection is necessary during the refeeding period.

Several limitations of this study need to be acknowledged. Due to the vulnerability of the patient group comprising premature infants, achieving a sufficient sample size through power calculation was difficult. Nevertheless, this was the first RCT for MFR that explored the benefits and safety of MFR with a controlled protocol. Hence, it can provide the basis for further RCTs for MFR.

## Conclusion

MFR for preterm infants with enterostomy is advantageous for growth and intestinal adaptation as shown by this exploratory RCT. MFR is relatively safe if performed using a standardized protocol with monitoring of infection. Future studies should investigate the infections that occur as MFR-related complications.

## Data Availability

The datasets used and analysed during the current study are available from the corresponding author at reasonable request.

## Abbreviations

PN: Parenteral nutrition
MFR: Mucous fistula refeeding
RCT: Randomized controlled trial
NICU: Neonatal intensive care unit
EN: Enteral nutrition
PMA: Postmenstrual age

## References

[1] Eicher C, Seitz G, Bevot A, Moll M, Goelz R, Arand J (2012). Surgical management of extremely low birth weight infants with neonatal bowel perforation: a single-center experience and a review of the literature. Neonatology.

[2] Rich BS, Dolgin SE (2017). Necrotizing enterocolitis. Pediatr Rev.

[3] Eaton S, Rees CM, Hall NJ (2017). Current research on the epidemiology, pathogenesis, and management of necrotizing enterocolitis. Neonatology.

[4] Koshinaga T, Inoue M, Ohashi K, Sugito K, Ikeda T, Tomita R (2011). Therapeutic strategies of meconium obstruction of the small bowel in very-low-birthweight neonates. Pediatr Int.

[5] Mayer O, Kerner JA (2017). Management of short bowel syndrome in postoperative very low birth weight infants. Semin Fetal Neonatal Med.

[6] Puppala BL, Mangurten HH, Kraut JR, Bassuk A, Shrock P, Benawra RS, Napier K (1985). Distal ileostomy drip feedings in neonates with short bowel syndrome. J Pediatr Gastroenterol Nutr.

[7] Elliott T, Walton JM (2019). Safety of mucous fistula refeeding in neonates with functional short bowel syndrome: a retrospective review. J Pediatr Surg.

[8] Lau EC, Fung AC, Wong KK, Tam PK (2016). Beneficial effects of mucous fistula refeeding in necrotizing enterocolitis neonates with enterostomies. J Pediatr Surg.

[9] Yabe K, Kouchi K, Takenouchi A, Matsuoka A, Korai T, Nakata C (2019). Safety and efficacy of mucous fistula refeeding in low-birth-weight infants with enterostomies. Pediatr Surg Int.

[10] Wong KK, Lan LC, Lin SC, Chan AW, Tam PK (2004). Mucous fistula refeeding in premature neonates with enterostomies. J Pediatr Gastroenterol Nutr.

[11] Goday PS (2009). Short bowel syndrome: how short is too short?. Clin Perinatol.

[12] Amin SC, Pappas C, Iyengar H, Maheshwari A (2013). Short bowel syndrome in the NICU. Clin Perinatol.

[13] Wessel JJ, Kocoshis SA (2007). Nutritional management of infants with short bowel syndrome. Semin Perinatol.

[14] Gause CD, Hayashi M, Haney C, Rhee D, Karim O, Weir BW (2016). Mucous fistula refeeding decreases parenteral nutrition exposure in postsurgical premature neonates. J Pediatr Surg.

[15] Al-Harbi K, Walton JM, Gardner V, Chessell L, Fitzgerald PG (1999). Mucous fistula refeeding in neonates with short bowel syndrome. J Pediatr Surg.

[16] Koike Y, Uchida K, Nagano Y, Matsushita K, Otake K, Inoue M, Kusunoki M (2016). Enteral refeeding is useful for promoting growth in neonates with enterostomy before stoma closure. J Pediatr Surg.

[17] DiBaise JK, Young RJ, Vanderhoof JA (2004). Intestinal rehabilitation and the short bowel syndrome: part 1. Am J Gastroenterol.

[18] DiBaise JK, Young RJ, Vanderhoof JA (2004). Intestinal rehabilitation and the short bowel syndrome: part 2. Am J Gastroenterol.

[19] Rabier D, Kamoun P (1995). Metabolism of citrulline in man. Amino Acids.

[20] Windmueller HG, Spaeth AE (1981). Source and fate of circulating citrulline. Am J Physiol.

[21] Peterson J, Kerner JA (2012). New advances in the management of children with intestinal failure. J Parenter Enteral Nutr.

[22] Rhoads JM, Plunkett E, Galanko J, Lichtman S, Taylor L, Maynor A (2005). Serum citrulline levels correlate with enteral tolerance and bowel length in infants with short bowel syndrome. J Pediatr.

[23] Haddock CA, Stanger JD, Albersheim SG, Casey LM, Butterworth SA (2015). Mucous fistula refeeding in neonates with enterostomies. J Pediatr Surg.

[24] Cole CR, Frem JC, Schmotzer B, Gewirtz AT, Meddings JB, Gold BD, Ziegler TR (2010). The rate of bloodstream infection is high in infants with short bowel syndrome: relationship with small bowel bacterial overgrowth, enteral feeding, and inflammatory and immune responses. J Pediatr.

[25] Wolf L, Gfroerer S, Fiegel H, Rolle U (2018). Complications of newborn enterostomies. World J Clin Cases.

[26] Pataki I, Szabo J, Varga P, Berkes A, Nagy A, Murphy F (2013). Recycling of bowel content: the importance of the right timing. J Pediatr Surg.

[27] Ghattaura H, Borooah M, Jester I (2022). A review on safety and outcomes of mucous fistula refeeding in neonates. Eur J Pediatr Surg.

